# vHIT Testing of Vertical Semicircular Canals With Goggles Yield Different Results Depending on Which Canal Plane Being Tested

**DOI:** 10.3389/fneur.2021.692196

**Published:** 2021-07-27

**Authors:** Louise Wittmeyer Cedervall, Måns Magnusson, Mikael Karlberg, Per-Anders Fransson, Anastasia Nyström, Fredrik Tjernström

**Affiliations:** Department of Otorhinolaryngology, Head and Neck Surgery, Clinical Sciences, Lund Skåne University Hospital Lund, Lund, Sweden

**Keywords:** vertical vHIT, gain values, vertical semicircular canals, RALP, LARP, interacoustics, otometrics

## Abstract

**Objective:** The use of goggles to assess vertical semicircular canal function has become a standard method in vestibular testing, both in clinic and in research, but there are different methods and apparatus in use. The aim of this study was to determine what the cause of the systematic differences is between gain values in testing of the vertical semicircular canals with two different video head impulse test (vHIT) equipment in subjects with normal vestibular function.

**Study Design:** Retrospective analysis of gain values on patients with clinically deemed normal vestibular function (absence of a corrective eye saccade), tested with either Interacoustics or Otometrics system. Prospective testing of subjects with normal vestibular function with the camera records the eye movements of both eyes. Finally, 3D sensors were placed on different positions on the goggles measuring the actual vertical movement in the different semicircular planes.

**Results:** In the clinical cohorts, the gain depended on which side and semicircular canal was tested (*p* < 0.001). In the prospective design, the combination between the stimulated side, semicircular canal, and position of the recording device (right/left eye) highly influenced the derived gain (*p* < 0.001). The different parts of the goggles also moved differently in a vertical direction during vertical semicircular canal testing.

**Conclusion:** The gain values when testing the function of the vertical semicircular canals seem to depend upon which eye is recorded and which semicircular plane is tested and suggests caution when interpreting and comparing results when different systems are used both clinically as well as in research. The results also imply that further research and development are needed to obtain accurate vertical semicircular canal testing, in regard to both methodology and equipment design.

## Introduction

Vestibular testing has, in the last two decades, experienced tremendous progress, and it is now possible to evaluate all five sensory receptors of the inner ear. The most easily available is the head thrust or head impulse test (HIT). Since its first description, the bedside head impulse test has become one of the most important and useful clinical tests when examining patients suffering from acute dizziness or vertigo (1). This bedside test consists of a quick low-amplitude rotation of the patient's head while keeping vision on a target, stimulating the semicircular canals in the plane of the head movement. When performed toward the side of a vestibular lesion, the eyes will lag due to reduced vestibular input. This will cause the gaze to follow the direction of the head, instead of being locked on the visual target. When perceived, a corrective saccade is generated, indicating the impairment (1). Although it is possible to assess the function of the vertical semicircular planes in a clinical situation as well (2), those canals are more frequently evaluated with a device measuring both eye and head velocity (3), commonly referred to as vHIT (video head impulse test). The outcome of the test is described as gain (comparing the velocities of the head and eye) and the commercially available systems do the gain calculation differently, which seemingly also has its impact on the results (4, 5). If a subject has a functional and intact vestibulo-ocular reflex (VOR), the gain should be close to 1.0, i.e., the eyes turn the same amount and at the same speed as the head and, thus, are able to maintain visual fixation. Some of the devices use goggles equipped with both a video camera recording the velocity of the eye and a sensor recording the movement of the goggles. The devices have been described and tested for their clinical usefulness (6, 7).

In our laboratory, we have access to two commonly used commercially available vHIT systems using goggles, Otometrics and Interacoustics. Clinically, we have noticed that the gain values of impulse tests of the vertical canals seem to differ between which of the planes being tested, i.e., right anterior–left posterior (RALP) seem to differ from left anterior–right posterior (LARP) both depending on which system was being used but also between measurements in the different planes compared with each other within the same equipment. The method of performing the head movements when examining vertical semicircular canals differ between the two systems. With Otometrics, the eyes should be aligned with the plane of movement and the head turned (8). With Interacoustics, the gaze should be directed straight ahead as well as the head, while performing the movement of the head in the plane of the semicircular canal. Both systems (i.e., both camera and accelerometer of the goggles) only detect vertical movement and not torsional movement, which is primarily the purpose as to why the Otometrics system has the gaze alignment in the plane of movement, to minimize torsion and produce vertical eye movements (8).

When performing clinical follow-ups, evaluating the use of different vHIT equipment, the findings obtained raised questions about why both the Otometrics and Interacoustics equipment seemingly produced systematically smaller gain values when the semicircular canals in the LARP plane were assessed compared with when the semicircular canals in the RALP plane were assessed. This study, therefore, had two aims; (1) To determine whether the two goggle-based vHIT equipment from Otometrics and Interacoustics produced, when evaluated in clinical contexts, systematically smaller gain values in the LARP plane compared with in the RALP plane. (2) To investigate whether any differences in gains between RALP and LARP planes might be explained by the placement of the recording devices on the goggles. Our hypothesis was that certain complex 3D movement trajectories of the head and goggles, and thus, of the recording device, might produce ambiguous data from which it was difficult to obtain a correct analysis. This effect might be manifested as systematic gain asymmetries when using different recording device positions.

## Materials and Methods

### Study Part 1, Presence of Systematic Right Anterior–Left Posterior/Left Anterior–Right Posterior Gain Asymmetries in Clinical Contexts

In study part I, data from two different equipment from the manufacturers Otometrics and Interacoustics were evaluated. Both equipment uses a similar approach, where a recording device containing different parts for simultaneously recording both eye movements and head movements is mounted on goggles attached to the head of the subjects while performing the head thrusts. Both pieces of equipment were evaluated in real clinical contexts on large representative patient populations that of various reasons were suspected to suffer from vestibular disorders (see Table 1). Both pieces of equipment were used exactly as detailed by the manufacturers. The calibrations and assessments were performed according to the specifics of each equipment. Two operators performed the examinations, about half of the examinations each, and they were trained on site by representatives from the manufacturers. As part of the prestudy evaluations, it was ensured that there were no systematic differences between the results obtained by each of the operators.

**Table 1:** The different diagnoses for the patients that performed video head impulse test (vHIT) with the different equipment.

**Diagnoses**	**Otometrics**	**Interacoustics**
Mb Menière	29 (30%)	9 (29%)
Vestibular neuritis (control)	7 (7%)	4 (13%)
Vestibular schwannoma	3 (3%)	4 (13%)
Vestibular migraine	5 (5%)	1 (3%)
PPPD	8 (8%)	3 (10%)
BPPV	9 (9%)	0 (0%)
Alternobar vertigo	1 (1%)	0 (0%)
Meningioma	0 (0%)	1 (3%)
Temporal bone fracture	1 (1%)	0 (0%)
Fistula	0 0%)	1 (3%)
Central vertigo	1 (1%)	0 (0%)
Cochlea implant	5 (5%)	0 (0%)
Dizziness	29 (30%)	8 (26%)

We retrospectively scrutinized the gain values for vHIT measurements deemed clinically as normal by an experienced neuro-otologist (MK, MM, FT). For qualifying as a normal HIT, the eye and head velocity recordings were manually analyzed, and even if abnormal gain (i.e., > or <1.0) was found, then the absence of corrective eye saccade indicated a functional VOR. Ninety-eight (98) consecutive vHITs were analyzed when Otometrics had been used (37M, 61F) aged 55 ± 15.6 (SD) years. For Interacoustics, 31 consecutive vHITs were analyzed (13M, 18F) aged 55 ± 15.0 years. The gain values used in this study were the ones the respective systems delivered on their result printouts. However, the methods used to calculate gain differ between the Otometrics and Interacoustics systems. With Interacoustics, it is a simpler calculation of the velocity of the eye movements divided with the velocity of head movements for each sample during 0–100 ms, upon which regression analyses are performed yielding a gain value (7). In Otometrics, the gain value is calculated by the use of “area under the velocity curves” for the eye movements and head movements, omitting any corrective eye saccade by signal processing (9).

### Study Part 2, Relationship Between Recording Device Placement on Goggles and Found Right Anterior–Left Posterior/Left Anterior–Right Posterior Gain Asymmetries

**Figure 1:**
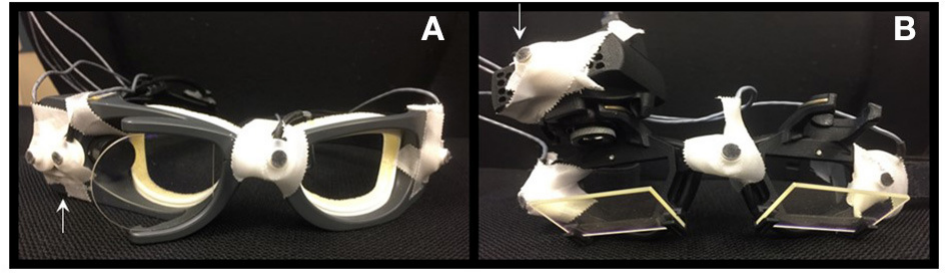
Sensor positions for Zebris measurements on Otometrics in **(A)** and for Interacoustics in **(B)**. The arrows indicate the positions of the recording devices attached to the googles.

The main purpose of performing study part 2 was to find reasons on how the systematic gain value deformations found in study part 1 clinical data could have been produced. In part 2, we examined 12 healthy volunteers (5M, 7F) aged 33 ± 10.8 years. vHIT with the Interacoustics system was performed. This system has the advantage of recording devices that can be mounted over both eyes simultaneously. Thus, by using two recording devices, one over the left eye and one over the right eye, it was possible to investigate whether the gain recorded differed between the two eye positions as perceived by the individual recording devices. It should be noted that both recording devices, each positioned above a different eye, simultaneously recorded the effect of the same head trust and, thus, whether the effect of this head thrust was recorded differently by the two devices (see **Figure 3**). The design of this control experiment was aimed to eliminate the potential influence from commonly suggested biasing factors. Before performing study part 2, it was ensured that the additional weight from one recording device would not affect the outcome. One subject was tested with two recording devices and then with one recording device twice on the different positions. The extra weight did not affect the gain values as shown in Supplementary Presentation 1 Figure 1. The average gain (i.e., the quotient between average eye velocity and head velocity) during the head acceleration phase (0–100 ms) based on linear regression of all impulses were analyzed.

### Study Part 3, Goggle and Recording Device Movements During Right Anterior–Left Posterior/Left Anterior–Right Posterior Trajectories

In study part 3, we put sensors on the goggles of the different systems and measured the horizontal movement (mm) in space using a Zebris© 3D ultrasound tracking system for motion detection. Thereafter, head movements were performed exactly according to the different specifications of Otometrics and Interacoustics on one subject.

### General Characteristics for Study Part 1 and Part 2

Both the Otometrics and Interacoustics equipment were used exactly as detailed by the manufacturers. The calibrations and assessments were performed according to the specifics of each equipment. All assessments in part 1 and part 2 were performed by two operators with long experience with both systems and to the specifics of hand placing and movement/alignment of the head. The software from the manufacturer set limits about how slow or fast a head movement should be to appropriately analyze the equipment. Thus, no head thrusts and results that were not approved by the software of the manufacturer were analyzed and included in study part 1 or study part 2. Recording device slippage can affect the gain values calculated, and thus, all recordings in study part 1 and part 2 were visually inspected prior to being included in the study.

### Procedures Used for Simultaneous Assessments With Two Video Head Impulse Test Recording Devices (Study Part 2)

All subjects were examined in study part 2 under identical conditions as follows. vHIT samples were made in a well-lit room. Prior to the sampling, calibrations were performed for both the two independent Interacoustics recording devices individually, first by asking the subject to focus on different positions using a five-point laser grid. Second, the subject was asked to do small, sinusoidal rotation of the head in the horizontal and vertical planes, with gaze focus remaining on a central target. Both calibrations and the subsequent data collections were performed using two different computers, one each per recording device. During the vHIT tests, the operator was standing behind the subjects, with hands placed on the subject's jaw to minimize goggle movement. Subjects were asked to keep visual focus on a target projected 1 m in front of them. Impulses were made in all semicircular planes. Impulses with peak head velocity <150°/s were automatically rejected by the vHIT software.

### Procedures Used for Assessment of Goggles and Recording Device Movement (Study Part 3)

The 3-D motion analyzer Zebris-CMS, with computer program WinSpine version 1.78 (Zebris Medizintechnik GmbH, Isny, Germany) was used with ultrasound markers taped on the goggles as depicted in Figure 1. The measuring unit was positioned on a stand approximately 1 m from the subject. The ultrasound microphone markers on the goggles received signals from the transmitters located in the measuring unit and were sampled by a computer at 50 Hz. The Zebris system analyzes position according to the principle of the timing of the interval between the emission and reception of ultrasound pulses. The absolute 3-D coordinates were calculated by triangulation. The subject was positioned on a chair, and the vertical head impulses were performed as specified by the different systems and the measuring method adjusted according to the different head positions. The results were recorded on a computer, and the maximum of the movements from 10 head impulses in the anterior plane of each LARP/RALP plane were measured. In order to limit the amount of presented data, only the data from the anterior planes are presented.

#### Statistics

Repeated-measures GLM ANOVA was used after ensuring that all analyzed dataset combinations produced model residuals that had normal or near-normal distribution, thus, validating the statistical method (10). The main factor combinations analyzed for their effects on the movement pattern from balance perturbations were:

(1) Stimulated side (right vs. left, df 1) and semicircular canal (lateral vs. anterior, df 1; lateral vs. posterior, df 1; anterior vs. posterior, df 1).
(2) Stimulated side (right vs. left, df 1) and semicircular canal (lateral vs. anterior, df 1; lateral vs. posterior, df 1; anterior vs. posterior, df 1) and recording device position (right vs. left, df 1).

In analysis 1, both model parameters, stimulated side and semicircular canal, are within-subject variables. In analysis 2, all model parameters, stimulated side, semicircular canal, and recording device position are within-subject variables.

In the *post-hoc* analyses, Wilcoxon matched-pair signed-rank test (Exact Sig two tailed) was used for analyzing the differences between the different canals of each ear in study part 1 and between the different recording device positions in study part 2. A Bonferroni correction was applied, and the significant *p*-value level was set to *p* < 0.025 in *post-hoc* tests and at *p* < 0.05 in the repeated measures GLM ANOVA. Nonparametric statistical tests were used in all *post-hoc* statistical evaluations since the Shapiro–Wilk test revealed that some data sets were not normally distributed, and normal distribution could not be obtained by log transformation.

#### Ethical Approval

The experiments were performed in accordance with the Helsinki declaration and approved by the local ethical board (Dnr 2016/32, EPN, Lund University, Sweden). All subjects gave their written and informed consents prior to participation.

## Results

### Study Part 1

**Figure 2:**
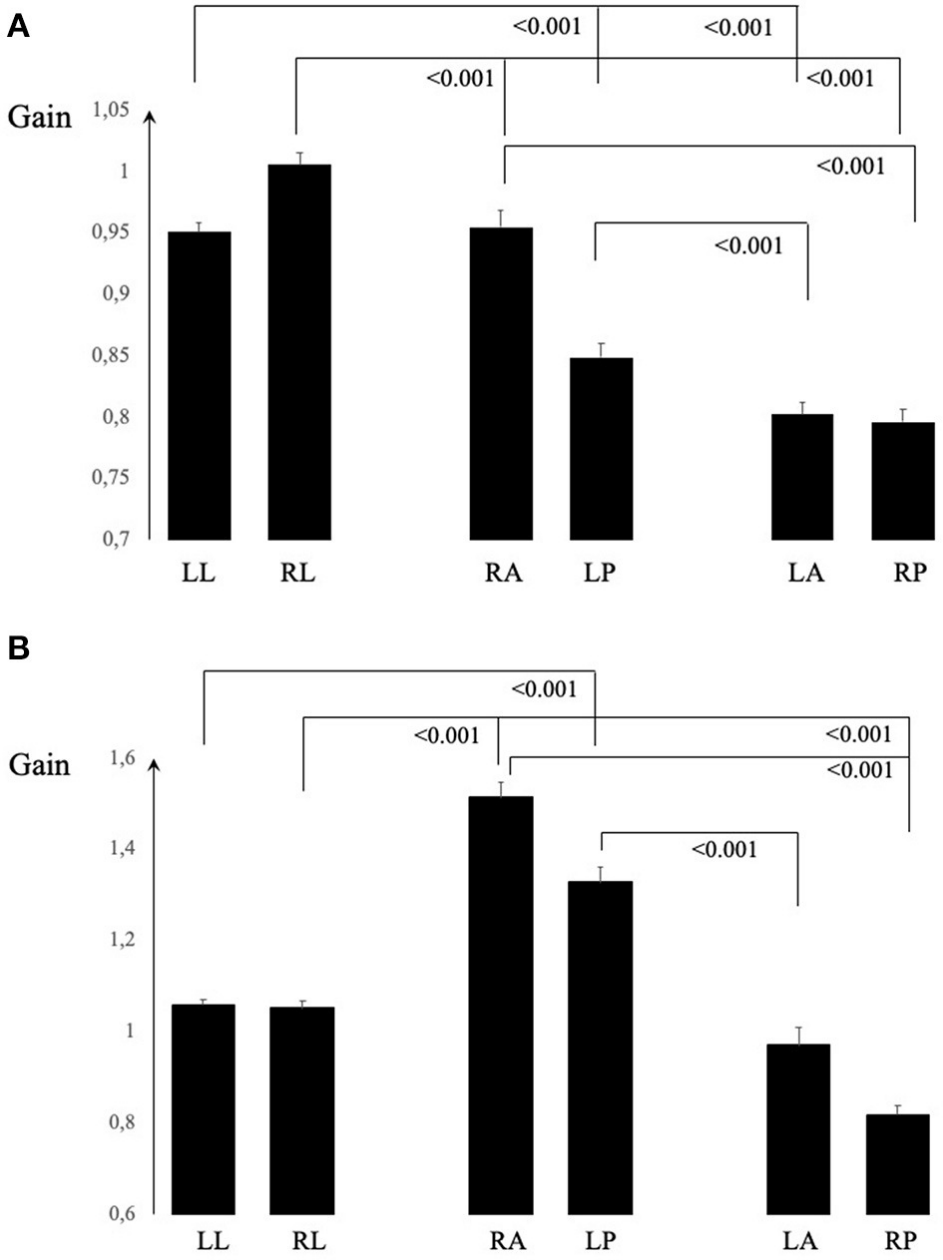
In **(A)**, the average and SEM gain values for Otometrics are shown and in **(B)** for Interacoustics. The values from each canal differed significantly from the two others in each ear tested for both equipment, except the left lateral from left anterior for Interacoustics equipment **(B)**.

The gain values from 98 consecutive vHIT recorded with the Otometrics system are demonstrated in Figure 2A and from 31 consecutive vHIT with Interacoustics in Figure 2B. GLM ANOVA statistical analyses are presented in Table 2. When assessed in a clinical context, the RALP/LARP gain asymmetries were of the scale between 6 and 20% (*p* < 0.001) for the Otometrics equipment and between 33 and 85% (*p* < 0.001) for the Interacoustics equipment. These found asymmetries in gain could not be explained or supported by other clinical findings, e.g., the presence of covert and/or overt saccades. For both pieces of equipment, the results differed significantly (*p* < 0.001) between the different semicircular canals, except in the lateral vs. posterior canal with the Interacoustics system. As can be seen in the figure, this was because the gain from the posterior canals were higher (left) and lower (right) than the lateral canal gain. The interaction analysis between the side and canal (Table 2) shows that the gain differs significantly for all semicircular canals (*p* < 0.001). For both systems, the gain values from the canals of each ear differed significantly (*p* < 0.001) except for Interacoustics between the left lateral and the anterior canal. As can be seen, the gain did not, in any canal or in any system, go below 0.8. The different diagnoses of patients involved are shown in Table 1.

**Table 2:** Repeated measures GLM ANOVA with main factors “stimulated side,” “semicircular canal,” and their factor interactions.

	**Otometrics**			**Interacoustics**		
	**Side right/left**	**Semicircular canal**	**Side x canal**	**Side right/left**	**Semicircular canal**	**Side x canal**
Lateral vs. anterior	<0.001 [141.9]	<0.001 [103.1]	<0.001 [32.3]	<0.001 [90.4]	<0.001 [29.4]	<0.001 [127.3]
Lateral vs. posterior	ns	<0.001 [389.9]	<0.001 [61.9]	<0.001 [161.9]	ns	<0.001 [87.0]
Anterior vs. posterior	<0.001 [48.3]	<0.001 [42.1]	<0.001 [73.1]	ns	<0.001 [94.9]	<0.001 [195.2]

*F-values are presented in the squared parenthesis. The table corresponds to Figures 2A,B*.

*ns means “not statistically significant”*.

### Study Part 2

**Figure 3:**
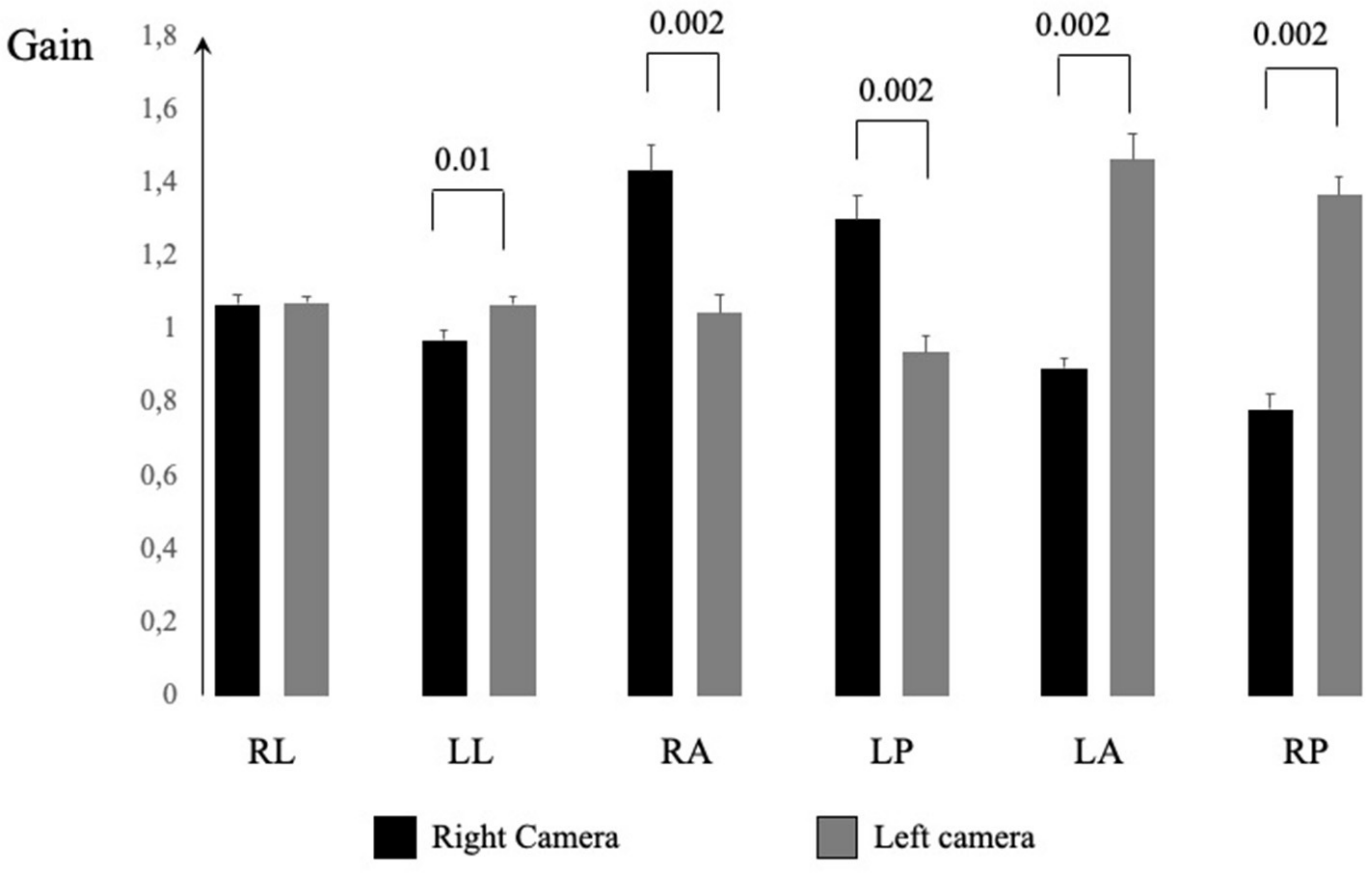
The average and SEM gain values from each canal and recording device positioned over the left or right eye.

**Figure 4:**
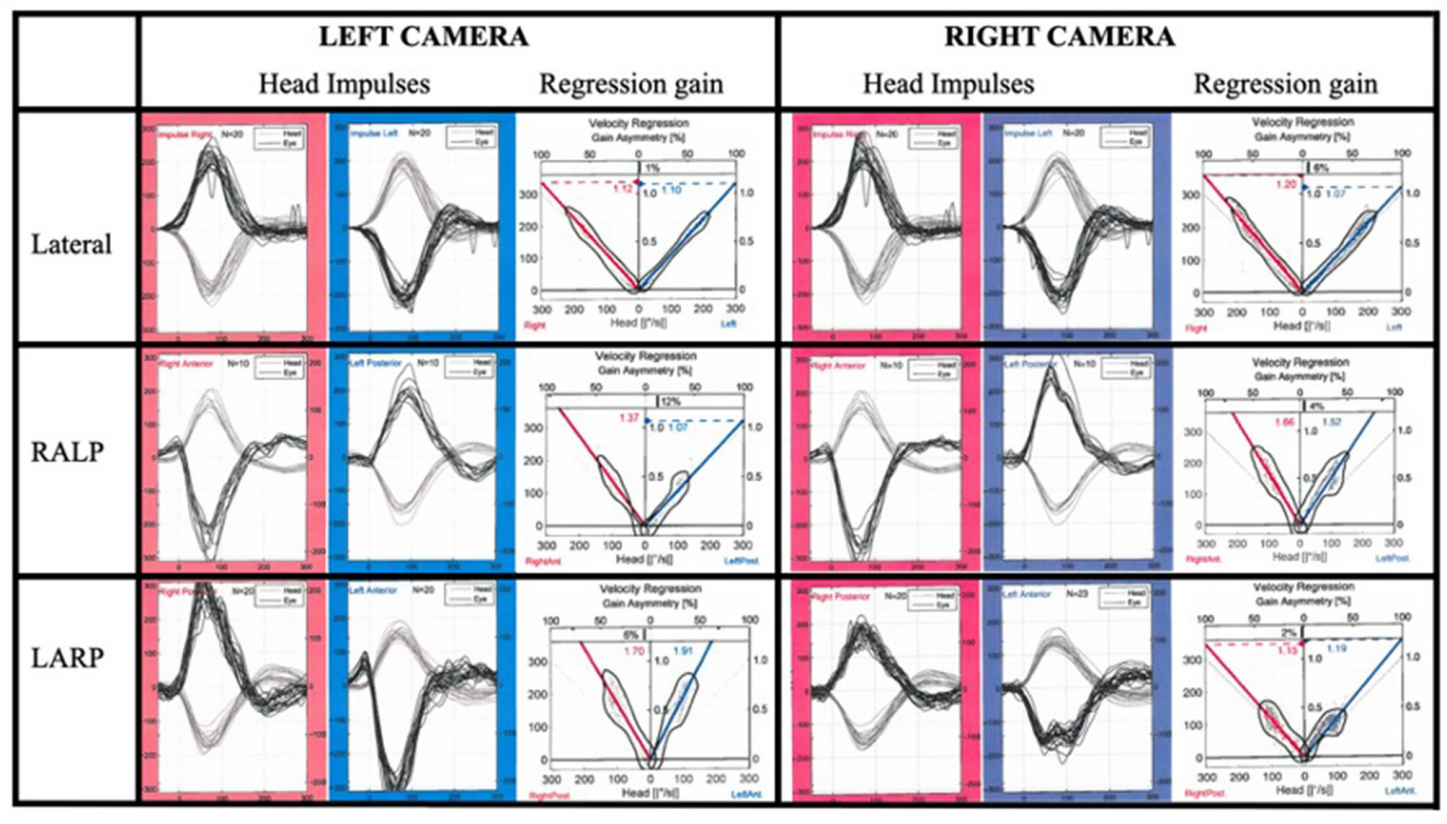
The generated eye and head velocities from one subject with all canal HITs recorded simultaneously by two devices positioned above different eyes.

In Figure 3, the gain values from each canal and right/left recording device placements are shown. The GLM ANOVA statistical analyses are presented in Table 3. Due to the asymmetrical gain values, there were no statistically significant differences between the stimulated side (right/left) and only occasionally depending on the semicircular canal or recording position. When analyzing the combination of all three factors, stimulated side, canal, and recording the device position, the gain differed statistically significant (*p* < 0.001) for all canals. *Post-hoc* analyses presented in Figure 3 show that the recording device position yielded significant differences in all canals, except when testing the right lateral canal. For each vertical canal, the position of the recording device yielded statistically significant different gains (*p* = 0.002). There were no significant differences in the lateral canal gain, but for each vertical canal, the differences were all significant (*p* = 0.002 or less). The figure also illustrates that if the right eye was monitored, the gain was higher in the RALP plane, and vice versa, the left eye recording device yielded higher gain in the LARP plane. In Figure 4, the raw data from one subject are shown.

**Table 3:** Repeated measures GLM ANOVA with main factors “stimulated side,” “semicircular canal,” and “recording device position” and their factor interactions.

	**Stimulated side right/left**	**Semicircular canal**	**Recording device position right/left**	**Side x canal**	**Side x recording device position**	**Canal x recording device position**	**Side x canal x recording device position**
Lateral vs. Anterior	ns	0.001 [24.8]	ns	ns	<0.001 [71.8]	ns	<0.001 [48.1]
Lateral vs. Posterior	ns	ns	0.009 [11.1]	0.023 [7.6]	<0.001 [57.5]	ns	<0.001 [69.5]
Anterior vs. Posterior	ns	0.005 [13.7]	ns	ns	ns	ns	<0.001 [74.6]

*F-values are presented in the squared parenthesis. The table corresponds to Figure 3*.

*ns means “not statistically significant”*.

### Study Part 3

**Figure 5:**
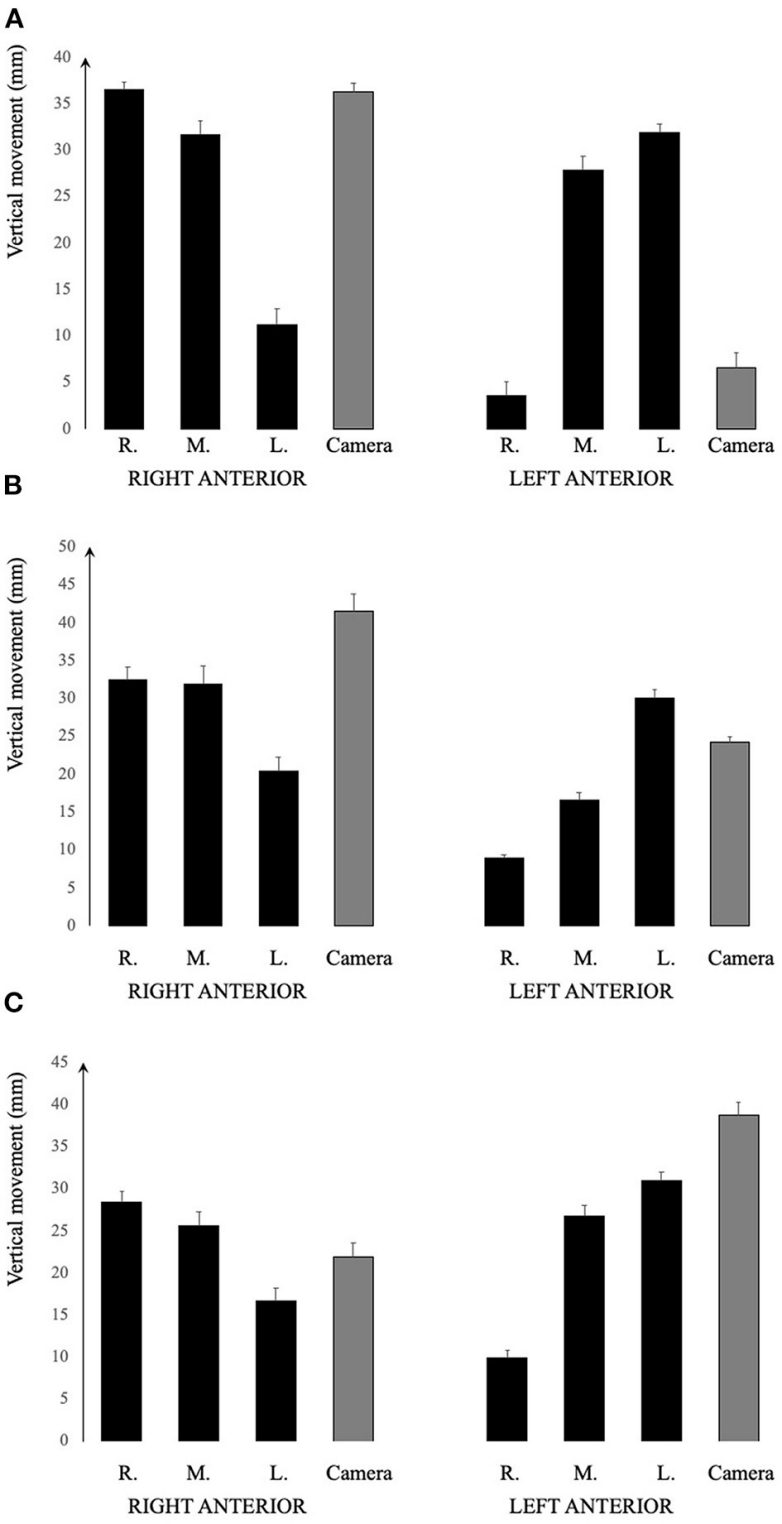
Graphical illustration of the vertical movement of the different positions of the goggles. **(A)** Otometrics. **(B)** Interacoustics recording device over the right eye. **(C)** Interacoustics recording device over the left eye. It would seem that the middle portion of the goggles moved more consistently far vertically, regardless of which canal plane tested. However, the sides of the goggles including the position of the recording device varied depending on the canal plane as well as on the position of the recording device.

In Figures 5A–C, the vertical movement in mm of the goggles are shown for movement in the anterior canal planes for the two different equipment and whether the recording device was placed over the right or left eye (Interacoustics). The amplitudes of the actual vertical movements of the different sides of the goggles varied depending on which canal plane was tested. In Supplementary Video 1, the actual vertical movements can be seen using the different equipment.

## Discussion

The gain values in the vertical semicircular canal planes were dependent upon which plane RALP/LARP was investigated, demonstrated both by the clinical material in study part 1 and the experimental material in study part 2. The main purpose of performing the control experiment in study part 2 was to find the reasons on how the systematic value deformations found in the clinical data (study part 1) could have been produced. We were able to reproduce the value distortions found in the clinical study in the study part 2 prospective study. The gain value distortions were related to over which eye the recording device was placed and to what kind of head movement trajectory was made. Hence, complex 3D movement trajectories of the head and goggles, and thus, of the recording device, might produce ambiguous data from which it is difficult to obtain a correct analysis. The gain values could be improved by changing the position of the recording device, i.e., examining RALP with the recording device over the left eye, and LARP with the recording device over the right eye. This is, so far, only possible to do with the system provided by Interacoustics. However, this also means that there has to be a second calibration, and thus, the examination will take more time as this is not included in the standard procedure recommended by the manufacturers. The reason why the gain is dependent upon the investigated canal plane seems at least partly to be due to the actual movement of the goggles and recording device in 3D space. As it is unclear where the motion sensor is placed in the goggles, for all but the manufacturers, the exact effect of how the different movements of the goggles affect the vertical motion used for calculating the gain is difficult to determine.

The two systems also have different ways to calculate gain, an issue that the current paper does not address, but it has been shown that also the outcome (gain) differs when using the same head and eye movements (11). Neither algorithms seem to consider the effect of the differences of the movement of the sensors as such, depending on which canal plane is tested and in which position the sensors are positioned and, if they do, then apparently not enough to compensate the differences in order to approach a gain value of 1.0.

Most of the research done on vHIT test reliability has been focusing on examinations of the lateral semicircular canals (4, 12). It is important to note that the gain and movement when examining the lateral semicircular canals in the present study were largely unaffected by the position of the recording device and did not differ neither in the clinical nor the experimental material. The test of lateral canals has shown a reliable test–retest and inter-examiner reliability (13). The vertical canals have also been examined to test test–retest reliability, with the result that vertical canals seem to yield less consistent values, even if one system seems to be superior than the other (13).

Clinical situations in which the vertical semicircular function would be important to assess encompass, but are not limited to, diagnosing an inferior vestibular neuritis (posterior semicircular canal) (14) or to assess remaining vestibular function prior to gentamicin treatment in Mb Menière (15) and in vestibular schwannoma patients (16). According to our clinical experience, both systems are able to properly find patients that have no vertical canal function, i.e., a very low gain combined with a corrective saccade. However, if a relative dysfunction is present, the results of the tests become more problematic. The movements of the head in the vertical planes, no matter the different specifications of the two systems at hand, are more difficult to perform and require practice and experience. Practice is needed in order to perform the movement correctly and equally important to conduct with sufficient velocity because if it is too slow, it is not certain that VOR is tested at all and that corrective saccades do not become apparent (6).

The findings in this study have a profound effect on how recordings and studies should be interpreted when assessed with standard vHIT equipment used today and also questions whether studies are comparable when using different systems. It is also clear from the present results that vHIT with the use of goggles in their present form do not reflect actual canal function and either that the techniques, equipment design, algorithms, or all together need further development. There is no question that the development of vHIT systems have furthered the clinical assessment of vestibular patients to a great extent. The market has today two different goggle-based devices with different specifications as to testing. Gaze alignment has been shown to be critical in eliminating torsional eye movements (which neither system can record) through elaborate search coil methods (17). In that study by Migliaccio and Cremer, the two different head movements (similar to that of Otometrics and Interacoustics) were compared with the different gaze alignments. When testing the vertical planes, the gain approached 1.0 when testing similar to the Otometrics specifications (i.e., gaze in the line of vertical movement), but the head coil was also moved according to the plane being stimulated—which would be similar to our experimental protocol and return values very much in line with our results. The gain algorithm, whatever way it is calculated, must also include a denominator value that does not change depending on which plane the head movement being conducted is in. It would, from our results, seem to be insufficient to measure the movements of the head from one fixed place on the goggle frame. It is vital to recognize the limitations of the examinations and let that drive a further development of the procedure as it is at present not satisfactory, not in any way diminishing previous efforts.

## Conclusion

Tests of vertical semicircular canals with vHIT yield different gains depending on which plane LARP/RALP is being tested and which eye is recorded. When investigated in clinical contexts, the gain asymmetries found were of the scale between 6 and 20% (*p* < 0.001) for the Otometrics equipment and between 33 and 85% (*p* < 0.001) for the Interacoustics equipment. Hence, the systematic asymmetries found were of a level where they will have clinical implications. A prospective study performed could attribute the gain asymmetries found to the use of goggles with fixed sensors and cameras. The gain value distortions were related to over which eye the recording device was placed and to what kind of head movement trajectory was made.

## Data Availability Statement

The raw data supporting the conclusions of this article will be made available by the authors, without undue reservation.

## Ethics Statement

The studies involving human participants were reviewed and approved by Etikprövningsmyndigheten, Lund University Sweden. The patients/participants provided their written informed consent to participate in this study.

## Author Contributions

FT, MK, MM, and P-AF conceived the study, interpreted results, and contributed to the manuscript. LW and AN executed the study, interpreted results, and contributed to the manuscript. All authors contributed to the article and approved the submitted version.

## Conflict of Interest

The authors declare that the research was conducted in the absence of any commercial or financial relationships that could be construed as a potential conflict of interest.

## Publisher's Note

All claims expressed in this article are solely those of the authors and do not necessarily represent those of their affiliated organizations, or those of the publisher, the editors and the reviewers. Any product that may be evaluated in this article, or claim that may be made by its manufacturer, is not guaranteed or endorsed by the publisher.

## Supplementary Material

Supplementary Table 1 Measured gain from each individual in study part 1 and 2.

Supplementary Video 1 Recording the vertical movements in space from different parts of the googles, while performing vertical movements as specified by the manufacturers, with Zebris© 3D ultrasound tracking system.

Supplementary Presentation 1 Figure 1 vHIT curves from one subject testing the vertical canals with one and two cameras respectively.
