# Supplementary figures and images for: vHIT Testing of Vertical Semicircular Canals With Goggles Yield Different Results Depending on Which Canal Plane Being Tested
Source: Front Neurol. 2021 Jul 27;12:692196. doi: 10.3389/fneur.2021.692196 (PMC8353365; doi:10.3389/fneur.2021.692196)

## Slide 1
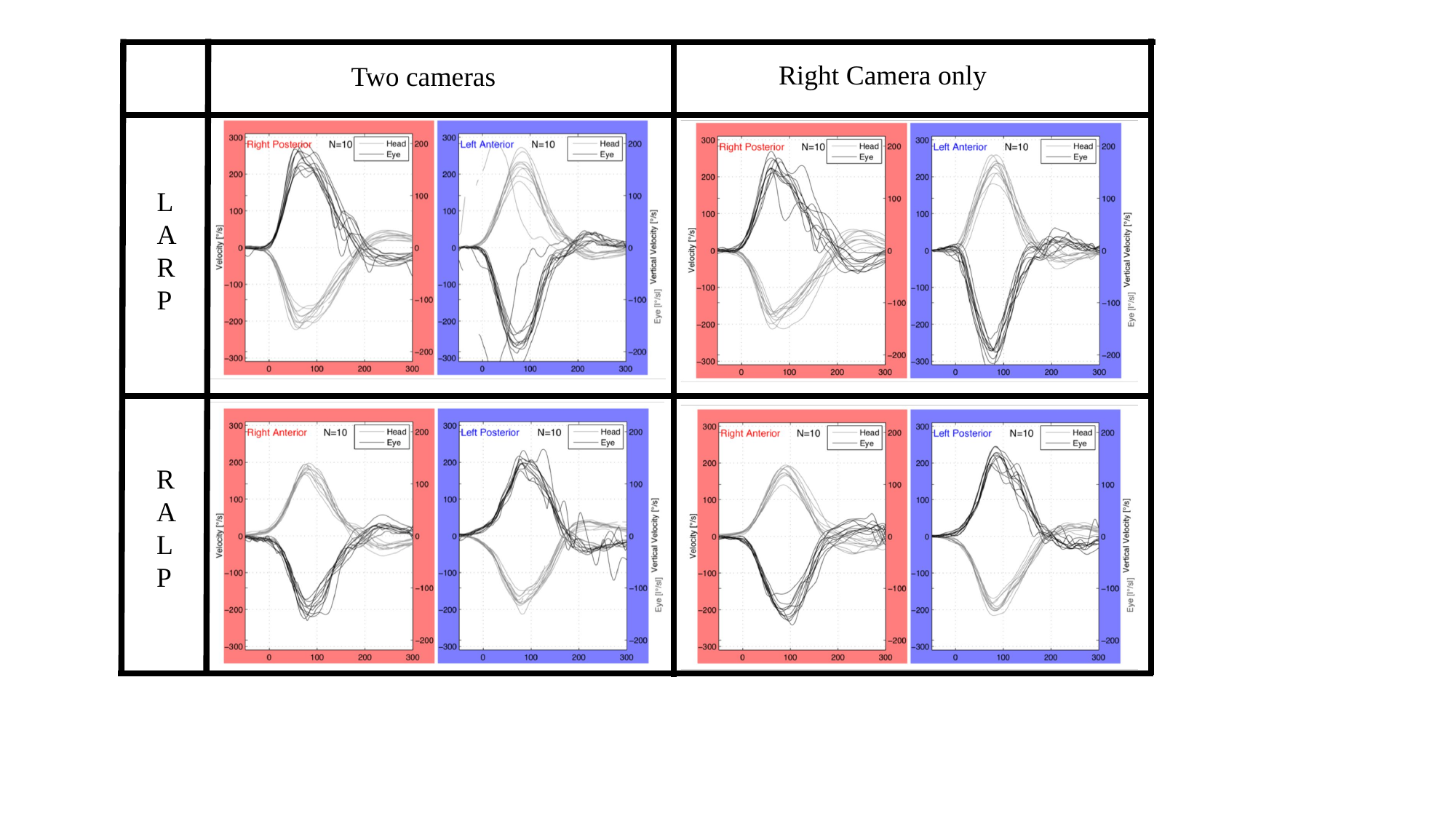

Right Camera only
Two cameras
L
A
R
P
R
A
L
P

Supplement: Supplementary Presentation 1 Figure 1 — vHIT curves from one subject testing the vertical canals with one and two cameras respectively. [file Presentation_1.PPTX]
